# That Case of Septic Salpingitis

**Published:** 1885-10

**Authors:** J. W. McLaughlin


### That Case of Septic Salpingitis.

*Dr. J. W. McLaughlin's Reply to Dr. Swearingen's Criticism.*

EDITOR DANIEL'S TEXAS MEDICAL JOURNAL:

IN the April number of the *Texas Courier Record of Medicine* I reported a case of septic salpingitis, involving the left oviduct, which followed parturition, and which was successfully treated by injecting a solution of mercuric bi-chloride, one grain to the pint, into the womb, in such a manner as that the contractions of this viscus would force the solution into the diseased tube. The treatment was based upon the hypotehsis that the solution was both aseptic and anti-septic; hence it would be as inocuous to the peritoneal cavity, if it should enter it, as the fluid in ascites; whilst its anti-septic properties would render it destructive to the pathogenous bacteria which it was assumed, had found a lodging place in the left fallopian tube, and had, as a result of their fermentive action, given rise to its inflammation, and the production of toxic agents, the absorption of which was seriously threatening the life of my patient.

The August number of your JOURNAL contains a criticism from the pen of Dr. R. M. Swearingen, upon the method adopted to reach the diseased tube, and its contained bacteria. He says:

"According to the programme, the womb had to be temporarily converted into a kind of *mitrailleuse* loaded with germicidal shot, and fired into a colony of bacteria." * * * "The object to be attained was good, the plan of attack ingenious, but could it be successful? The pus was confined in the left fallopian tube; the mouth of that tube was closed, and had to be violently opened before the bi-cloride solution could enter.

When this was done, the injected solution came in contact with another fluid that occupied and filled to its utmost capacity, the narrow tube. Both fluids could not occupy the same space at the same time, nor could they possibly mix. When the solution was driven in at one end of the tube, the pus, by molecular force, must have been as violently driven out at the other end, or the tube was necessarily ruptured. The conclusion then, seems inevitable, that the bi-chloride solution, when pressed by the contracting uterus, would have passed out through the *right* fallopian tube where no serious obstructions were met, instead of into the *left* tube which was hermetically closed. Nature is very obstinate, and will not violate a fixed law to accomodate a theory."

Reasoning from the Doctor's standpoint, the conclusions he deduces, are correct. In *poetry*, where great latitude is allowed the imagination, it would, perhaps, be the proper thing to compare the uterus to a *mitrailleuse* which fires bacterial shot through inelastic tubes. If we accept this simile, it seems evident that if one of these tubes is "filled to its utmost capacity" with its end "hermetically sealed," while the other is open and empty, the result would be,first,the "bacterial shot" would escape through the unobstructed tube, without breaking the seal or disturbing the contents of the other tube; or, second, the seal would be broken by the entering shot, (fluid) which would result in driving the contents of the tube out of its distal end.

Either hypothesis would be fatal to the theory of treatment adopted in the reported case of salpingitis, and would convict me of being ignorant of the rudimentary principles of physics. If this was a matter of pure imagery, I would recognize the poet's licence, and this reply would not have been made. But, in questions of science, facts must govern everything. The imagination cannot be allowed to wander at will, but must conform to, and be guided alone by facts.

In the light of the anatomy of the uterus and the fallopian tubes, and their behavior in certain pathological conditions, I think it will be shown that the Doctor's cast-iron hypothesis is wrong, throughout; wrong in its conception, and necessarily wrong in its conclusions.

The uterus is a hollow,pear-shaped viscus,composed of smooth muscular fibres, lined by a mucous membrane, and partially covered above by the peritoneum. The fallopian tubes are about four inches in length; are composed of three coverings, viz: peritoneal, muscular, and mucous, and for convenience, are divided into three portions, viz: interstitial, whence the mucous and muscular tissue of the tube blends with, and are continuous with those of the uterus; an abdominal part, and the ampulla, which terminates in the fimbræ, and open directly into the cavity of the peritoneum. The general contour of the tube is likened to that of a shepherd's crook; whilst its lumen is funnel shaped. At the intestitial end it will barely admit a small bristle; but widens from within outwards, and is longest in the ampulla. Now, the small calibre of the interstitial part of the tube, which will barely admit a bristle, becomes *less*, directly, as the womb diminishes in size, under muscular contraction; so that it is a question of serious doubt whether fluids can be forced through the womb into the peritoneal cavity, *in the living subject when the organs are in a state of health.* Heinrich Fritsch("Diseases of Women")expresses the opinion that it cannot be done. In the dead subject, however, where the muscular contractility and tonicity are destroyed; or in the living subject where inflammation of the parenchyma of the womb or fallopian tubes has brought about the same result, fluids can readily be forced through the womb, and into the peritoneal cavity.

The principle properties of muscular fibre are its contractility and tonicity. It is well known that septic inflammation of muscular tissue greatly impairs these properties; for instance, in acute septic metritis, the uterine canal is relaxed, patulous and readily dilatable. In septic inflammation of the oviduct this same condition would exist. At the uterine end of such oviduct, where the muscular fibres of the tube blend, and are continuous with the muscular fibres of the uterus, there would be a zone of inflammation surrounding the fallopian opening. Within this zone there would be paresis of the muscular tissue. As a result of this condition the opening of the duct would be relaxed, patulous, and dilatable.

Where inflammatory products accumulate in an inflamed oviduct, they would gravitate to its ampulla, which is its largest portion. If the fimbræ become united by adhesive inflammation, such products may accumulate in considerable quantity, provided their escape into the uterus is prevented by viscid mucus. The occurrence of adhesive inflammation at the ovarian end of the tube rather than at its uterine end is readily explained. The peritoneal sides of the fimbræ, in a state of inflammation, are in opposition, and bathed in fibrin,a highly organizable material. At the other end we have the mucous surfaces separated by mucus, which does not readily organize, and hence is a protection against adhesions of opposing surfaces.

When it is considered that the oviduct is an elastic tube, capable of great distension, (as in tubal dropsy) in some cases to the extent of containing a matured fœtus, it is absurd to say that two or four ounces of fluid will fill it "to its utmost capacity." This is a part of the Doctor's cast-iron hypothesis in which the tube is assumed to be absolutely inexpansible. He says: "Both fluids could not occupy the same space at the same time, nor could they possibly mix. When the solution was driven in at one end of the tube, the pus, by molecular force, must have been as violently driven out at the other end, or the tube was necessarily ruptured."

In the light of the above facts, anatomical and pathological, I ask what were the conditions of the uterus and tubes in the case of septic salpingitis which I reported? And what were the results of the intra-uterine injection used? The following facts indicate that the uterine mucosa had entirely recovered from the primary septic inflammation, viz: the constitutional symptoms indicating this condition, as pyrexia, rigors, pain, etc., had disappeared, and remained absent for eleven days prior to second invasion. Upon examination, I found the uterus, which had been previously enlarged, relaxed and flaccid, contracted, and much reduced in size; the cervical canal,(which had been patulous) was found so contracted, that I had difficulty in introducing the gum catheter through which the anti-septic fluid was injected.

The left tube was thought to be the place of infection, because the recurring rigors, fever, and other alarming symptoms which came up after the eleventh day, clearly indicated a new invasion of septic trouble. In the absence of symptoms which would locate the trouble in the uterus, and, in view of the fact that pain, tenderness and tension were felt in the region of the left oviduct, together with an area of dullness, indicated by percussion, led me to believe this tube to be the location of the trouble. Upon this diagnosis, I injected the fluid into the uterine cavity, expecting that the contractions of the womb would force it into the left oviduct, where it would mix with, and render aseptic, any fluid with which it would come in contact, and by rendering such fluid less viscid and dense, would cause it more readily to escape. I felt justified in this expectation, because the same contraction that would dilate the diseased tube would contract the sound one, and thereby prevent the fluid from escaping through it. The results were in perfect harmony with this theory. The day following the injection the patient passed about four ounces of pus from the uterus, the fever abated, rigors ceased, and she rapidly and fully recovered her health. Very respectfully,

J. W. McLaughlin.

